# The Quest for an HIV-1 Vaccine Adjuvant: Bacterial Toxins as New Potential Platforms

**DOI:** 10.4172/2155-9899.1000225

**Published:** 2014-07-17

**Authors:** Toufic O Nashar

**Affiliations:** College of Veterinary Medicine, Nursing & Allied Health, Department of Pathobiology, Tuskegee University, Tuskegee, AL 36088, USA

**Keywords:** Adjuvants, Enterotoxins, LT, LTB, EtxB, CT, HIV-1, ENV, Vaccines

## Abstract

While tremendous efforts are undergoing towards finding an effective HIV-1 vaccine, the search for an HIV-1 vaccine adjuvant lags behind and is understudied. More recently, however, efforts have focused on testing adjuvant formulations that can boost the immune response and generate broadly neutralizing antibodies to HIV-1 ENV (gp160). Despite this, there remain a number of challenges towards achieving this goal. These include safety of adjuvant formulations; stability of the incorporated antigens; maintenance of ENV immunogenicity; optimal inoculation sites; the effective combination of adjuvants; stability of ENV neutralizing epitopes in some adjuvant formulations; mucosal immunity; and long-term maintenance of the immune response. A new class of adjuvants for HIV-1 proteins is suggested to overcome many of the limitations of some other adjuvants. Type 1 (LT-I) and type 2 (LT-II) human *E. coli* enterotoxins (HLTs) and their non-toxic B-subunits derivatives are strong systemic and mucosal adjuvants and effective carriers for other proteins and epitopes. Their stable molecular structure in the presence of fused proteins and epitopes, and their ability to target surface receptors on antigen presenting cells make them ideal for the delivery of HIV-1 ENV or HIV other proteins. Importantly, unlike some other adjuvants, HLTs and derivatives have well-defined modes of immune system activation. The challenges in finding optimal HIV-1 vaccine adjuvant formulation and the important properties of HLTs are discussed.

## Challenges in the selection of an HIV-1 vaccine adjuvant

Diligent efforts are currently being made in the search of an HIV-1 vaccine adjuvant although this has not been at pace with efforts in finding a vaccine against HIV-1. There were probably good reasons for the slow progress. Besides the disappointing earlier clinical trial with recombinant ENV protein (VAX 003, VAX 004) and lack of numerous accessible neutralizing epitopes on the protein, which in the majority of cases are shielded by glycans, much needed to be understood about the intrinsic properties of the immunogen [1–3] before finding a suitable adjuvant. These included finding suitable conformation of the trimeric ENV protein that could stimulate broadly neutralizing antibodies, and the nature of epitopes that form the basis for the strong and broad neutralizing ability of some anti-HIV-1 polyclonal and monoclonal antibodies. The current interest in finding suitable adjuvants may have been accelerated by the findings that certain antibody responses correlated with protection from HIV-1 acquisition in the recent RV144 phase III trial. Adding to this is the discovery of a series of potent and broad spectrum neutralizing antibodies that have been previously isolated from infected individuals [4]. In the RV144 trial, antibodies correlated with reduced risk from HIV-1 infection. Thus, currently the challenge is to mimic those antibodies in a vaccine against HIV-1. Generating broadly neutralizing antibodies either synthetically or following immunization would have tremendous impact on HIV-1 infection either therapeutically to lower viral load in infected individuals, or in the prevention of HIV-1 infection, respectively. However, to achieve these goals a greater understanding of suitable adjuvant platforms for the vaccine is required. A number of challenges exist in this regard. These include: 1- The issue of toxicity of some adjuvants such as some cytokines or adjuvants not suitable for use in human. 2- Stability of the desired conformation of ENV in some adjuvant formulations [5]. 3- The site of inoculation where ENV and adjuvant work best [6]; these include subcutaneous, intramuscular, intranasal or oral. 4- A large dose of ENV is required for immunization [7]; hence, an adjuvant that results in a strong humoral immune response is required. 5- Appropriate combination of adjuvants might be required to enhance immunogenicity of ENV [8]. 6- Selection of adjuvant formulations that do not result in destruction of the broadly neutralizing epitopes before binding to the B cell receptor [9]. 7- Finding adjuvants that do not alter antigen processing of the neutralizing ENV epitopes in a way that alters re-elicitation of the same type of antibodies to the initial epitope. 8- Selection of the right combination of adjuvant formulations and inoculation sites in prime/boost regimens. 9- Finding suitable adjuvants that incorporate ENV fragments and maximize responses to neutralizing antigenic determinants in the absence of effects from non-essential dominant epitopes. 10- Finding adjuvant formulations able to induce high level of protective mucosal IgA antibody [10]. 11- Finding appropriate adjuvants able to stimulate T cell-mediated immunity to HIV-1 proteins or epitopes other than ENV namely, HIV-1 Gag, Pol and Nef [8, 11, 12].

## *E. coli* Heat-Labile Enterotoxins as Potential Adjuvants

**Figure 1:**
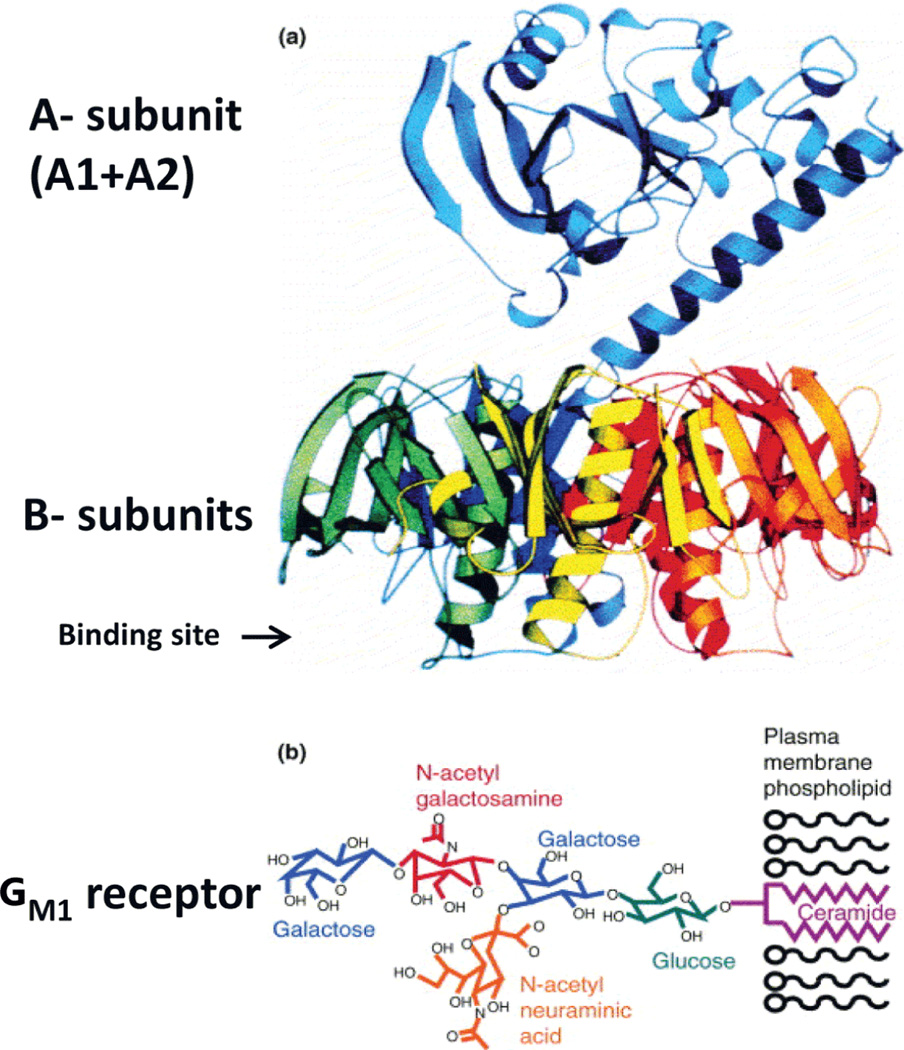
The molecular structure of HLT-I [similar to HLT-II except for the type of receptor (55)]. The family of LT and LT-II toxins contains an enzymatically active A1 domain and the A2 domain that allows for non-covalent interaction of the A subunit and the B-subunit pentamer to give holotoxin. Importantly, this molecular structure can be exploited to engineer chimeras with other proteins or epitopes. In the native structure of the holotoxin, A1 points away from the A2-B pentamer complex. Inserts can be engineered at the N terminus of A2 subunit to replace A1 (responsible for toxic activity) with the target of interest and thus still allow appropriate assembly. Such chimeric proteins assemble, do not interfere with the immunological function of the binding subunits of the toxins, and are non-toxic. Alternatively, fusions can be made directly to the C-terminus of the B subunits in the absence of the A subunits (A1 and A2). The binding site of the B subunits cross-links five moieties of G_M1_ via interaction with their pentasaccharide moieties exposed upon the lymphocyte cell surface. G_M1_ is integrated into the plasma membrane through its hydrophobic ceramide moiety. Diagram: a) A1/A2-B5 holotoxin; b) G_M1_ receptor.

To be highly effective, adjuvants should trigger a multitude of biological processes in antigen presenting cells (APCs) and be able to direct the immune response to relevant epitopes. A new class of bacterial toxins adjuvants may prove to be highly effective in priming the immune response to HIV-1 ENV, Gag, Pol and Nef proteins or derived epitopes. This refers to the family of type 1 (LT-I) and type 2 (LT-II) human *E. coli* enterotoxins (HLTs). HLTs contain an enzymatically active A1 domain responsible for toxicity, and the A2 domain that allows for non-covalent interaction of the A subunit and the non-toxic B-subunit pentamer to give holotoxin (Figure 1). LT-I, LT-II and their non-toxic B subunits derivatives modulate immune responses to other antigens by a number of mechanisms. These include effective targeting of fused proteins and epitopes to surface of APCs, alteration of cytokine production towards either T helper I (Th1), T helper II (Th2) or both, increased expression of co-stimulatory molecules on APCs, and expansion of T cells [13–19]. Recently, we demonstrated the potential role of LT-I nontoxic B-subunits in APC targeting and induction of T cell responses to HIV-1 gag p24 [20]. Many of the stimulatory effects of HLTs and their derivatives to other proteins are attributed to binding to surface receptors, such as gangliosides and Toll-like receptor 2 (TLR-2). Thus binding of LT-I to ganglioside G_M1_ receptor [13, 16, 17, 21], a component of lipid rafts, directly activates B cells [14] by increased levels of PI3K and MAP/ERK kinases [22]. The outcome of these signals is an upregulation of co-stimulatory molecules including MHC class II, B7-2, CD25, CD40 and ICAM-1 [14]. Non-toxic derivatives of LT-I also act on dendritic cells for stimulation of CD4^+^ T cells and secretion of cytokines [25, 26], and potentiate antigen- or virus-specific CTLs (23–25) independent of IL-12 and IFN-γ(24). Unlike CpG1826, non-toxic mutants of LT-I enhance germinal center reaction and prolong persistence of antibody-secreting cells in the bone marrow [26], properties that may be essential in broadening antibody specificities and memory to HIV-1 ENV. Further, LT-I or LT-IB subunits can be used to adjuvant a variety of soluble antigens [25, 27, 28], and plasmids encoding these molecules are strong adjuvants for the weakly immunogenic DNA vaccines [19]. Targeting of LT-IB fusion proteins to G_M1_ on APCs significantly enhances their presentation to T cells and immunogenicity [16, 21]. These findings are explained by the ability of LT-IB to deliver antigen cargo to MHC-I and MHC-II compartments [16, 29], and to a depot effect [21] in the APCs. Non-toxic mutants of LT-I conjugates also boost immune responses to a variety of polysaccharides [30, 31] while DNA vaccines are unable to express these molecules. Further, HLTs and recombinant fusions can be expressed in a variety of hosts including bacteria, yeast and plants [32–34]. In comparison to LT-I, research in Terry Connell laboratory (University of Buffalo, NY) demonstrated unique properties of LT-II and their derivatives. There are three types of LT-II namely, LT-IIa, LT-IIb and LT-IIc [18, 35] wherein LT-IIxB designates their B subunits pentamers. LT-IIaB binds to Toll-Like Receptor 2 (TLR-2) on mouse and human monocytes and induces secretion of TNF-α, IL-1, IL-6 and IL-8 by activation of NF-κB [36]. In contrast to LT-1, LT-IIaB upregulates expression of CD80 but not CD86 on mouse B cells [36]. LTIIaB also acts on dendritic cells by increasing their migration in nasal mucosa by upregulation of CCR7, enhancing uptake and presentation of co-administered antigen, and inducing maturation of dendritic cells by increased expression of CD80, CD86, and CD40 [36]. LT-IIaB effects on dendritic cells and other APCs occur following binding to TLR-2 [36]. LT-IIaB also augments antigen-specific CD4^+^ T cells proliferation, IgA and IgG antibodies [36].

Despite that HLTs can exert their adjuvant function both in a mixture or when conjugated to other proteins, there are more advantages in using chemical or genetic fusions [20]. These include stability and more efficient targeting of epitopes into pathways of antigen processing and presentation. The ease of HLTs genetic manipulation can be exploited to engineer chimeras with HIV epitopes either by using their non-toxic B subunits or modified holotoxins that have reduced or no toxicity. Both strategies have been proven successful with a number of proteins [37–40]. For LT-I, these include fusing an epitope of the *Bordetella pertussis* p69 antigen to LT-IB [39]. Fusions to LT-IIa and cholera toxin of a large protein, the saliva-binding region from the streptococcal adhesin AgI/II (SBR, ~ 42KD) were also made [40, 41]. Other chimeras include MBP of *E. coli*, HBsAg of hepatitis virus, and a surface antigen of *Haemophilus influenzae* (Connell, personal communication). Chemical conjugations of antigens to LT-IB and to the structurally and functionally related cholera toxin (CT) or cholera toxin B subunits (CTB) were also made and shown to boost immune responses to the coupled bacterial proteins and provide protection against the pathogens [42–47]. These include conjugation to CTB of a streptococcal surface protein (*S. mutans* antigen), ovalbumin, and keyhole limpet hemocyanin (KLH), and conjugation of CT to Sendai virus.

In other laboratories, bacterial toxins were used to boost immune responses to HIV-1 antigens. In some cases, the A1 subunit was used as an adjuvant combined with HIV-1 gag or HIV-1 gp120 [48]. CTB was also used as a scaffold for the V3 loop of HIV-1 gp120 [49]. In addition, two copies of the D-fragment of *Staphylococcus aureus* protein A fused to CTA1 (CTA1-DD) was also used to adjuvant monomeric and trimeric HIV-1 gp120 [50]. Moreover, CTB was fused to drug escape variant of HIV reverse transcriptase epitope [51]. In all these strategies, components of cholera toxin were shown to adjuvant immune responses to HIV-1 proteins.

The structural and functional properties of LT-I and LT-II and their derivatives will provide several advantages to ENV epitopes. The pentameric structure of the B subunits (Figure 1) allows fusions of 5 moieties of the desired epitopes thus maximizing the dose of antigen delivered. The toxic A1 subunit can be removed and replaced by ENV epitopes fused to the A2 subunit thus pointing away from the receptor binding site of the B subunits (Figure 1). This strategy has been successfully used with other proteins [40, 41]. Alternatively, fusions of the ENV epitopes can be engineered in the absence of the A1 and A2 subunits either at the C- or N-termini of the B subunits. The advantage of the latter approach is that it generates recombinants that allow fusion of 5 moieties of the desired epitopes. An additional approach would be to engineer ENV epitopes to mutants of the holotoxins (A1+A2+B) that have reduced or no toxic activities [52–54]. However, due to the large size of the hexameric ENV protein, a reductionist approach should be made by for example engineering monomeric fragments or fragments containing several B cell and Th epitopes so that the structural integrity of the toxins and their receptor binding site are preserved. Alternatively, chemical fusions could be generated with trimeric gp120 or gp41 or hexameric ENV to test their immunogenicity. Finally, different HIV epitopes including ENV antigens each loaded to either HLT-I or HLT-II can be used in a mixture to boost immunity against HIV.

In conclusion, HLTs may overcome several issues associated with the use of ENV and other HIV proteins in some adjuvant formulations. These include lack of stability of the antigens, reduced immunogenicity, limited use of inoculation sites (HLTs are effective at mucosal and in systemic compartments), destruction of neutralizing epitopes by adjuvants, inability to target epitopes to surface receptors on APCs, inability to induce long-term memory, and failure to generate quantitative and qualitative immune responses that can be maintained (Th1, Th2 or both). They may hold a great promise as adjuvants and carriers for HIV-1 proteins and epitopes particularly when used in combination with recombinant viral vaccines expressing HIV-1 proteins in prime/boost regimens.
